# Biomonitoring of bisphenol A, S, and F in urine samples from children in Finland

**DOI:** 10.1007/s10661-026-15401-2

**Published:** 2026-05-07

**Authors:** Parinaz Poursafa, Jani Koponen, Panu Rantakokko, Ida Helotie, Meri Koivusalo

**Affiliations:** 1 Faculty of Medicine and Health Technology, Tampere University, Tampere, Finland; 2 Finnish Institute for Health and Welfare (THL), Lifestyles and Living Environments Unit, Kuopio, Finland; 3 Faculty of Social Sciences, Tampere University, Tampere, Finland

**Keywords:** Human biomonitoring (HBM), Endocrine disruptor, Bisphenols, Children, Exposure, Risk assessment

## Abstract

Bisphenol A (BPA) and its analogues bisphenol S (BPS) and bisphenol F (BPF) are widely used in consumer products and linked to endocrine-disrupting effects. Young children may be especially vulnerable. This study assessed urinary concentrations of BPA, BPS, and BPF in Finnish children and evaluated health risks using biomonitoring guidance values and exposure modeling. First-morning urine samples were collected from 40 children aged 3–6 years in Tampere, Finland. BPA, BPS, and BPF were quantified using triple quadrupole mass spectrometry. Estimated daily intakes (EDIs) for BPA were derived using a physiologically based pharmacokinetic (PBPK) reverse dosimetry approach. Risk characterization was performed using Human Biomonitoring Guidance Values (HBM-GVs), interpreted in the context of the 2015 and 2023 EFSA tolerable daily intake values. BPA was quantifiable in 32.5% of samples and BPS in 15%, while BPF was not detected. Estimated BPA intakes (0.015–0.474 µg/kg bw/day) exceeded the 2023 EFSA TDI (0.2 ng/kg bw/day) by factors of 74–2,370. Under the updated TDI, all measured concentrations would exceed thresholds. For BPS, the maximum concentration (2.3 ng/mL) exceeded its HBM-GV (1 ng/mL), while no updated EFSA TDI is available. This study provides the first biomonitoring data on BPA, BPS, and BPF in Finnish children. BPA levels were lower than those reported in recent European studies, suggesting declining exposure trends. However, updated health-based benchmarks indicate that BPA intakes exceeded the 2023 EFSA TDI, while BPS levels exceeded its HBM guidance value. These findings highlight the need for continued biomonitoring and updated guidance values.

## Highlights

- First biomonitoring of BPA, BPS, and BPF in Finnish children’s urine
- Triple quadrupole LC–MS/MS enabled simultaneous quantification of urinary bisphenols
- BPA intakes exceeded 2023 EFSA TDI in all quantifiable samples based on PBPK modeling
- BPS exceeded HBM-GV in 7.5% of children; BPF was not detected
- Updated health benchmarks indicate ongoing bisphenol exposure concern in children

## Introduction

Bisphenol A (BPA) is a widely used chemical in the production of plastics and epoxy resins, found in consumer products such as food containers, water bottles, toys, electronics, and thermal paper receipts (D. Chen et al., 2016). As an endocrine-disrupting chemical (EDC), BPA interferes with hormonal functions, posing potential risks to children whose developing systems, particularly the brain, are highly susceptible (Huang et al., 2017; Lehmler et al., 2018; Louro et al., 2019; Lucarini et al., 2020). Early exposure to BPA has been linked to cognitive impairments, behavioral issues, and metabolic disorders such as obesity (Ejaredar et al., 2017; Kim et al., 2019). Children, especially toddlers, face heightened exposure through mouthing activities and contact with items like toys, carpets, and floors, making environments like daycare centers a key concern due to prolonged exposure to such materials (Dalamaga et al., 2024).

The growing evidence of BPA’s adverse effects has prompted regulatory action. In 2017, the European Chemical Agency (ECHA) classified BPA as a Substance of Very High Concern (SVHC) due to its impacts on reproductive, cognitive, and metabolic functions (Beausoleil et al., 2018). In 2023, the European Food Safety Authority (EFSA) reduced the tolerable daily intake (TDI) from 4 µg/kg body weight/day to 0.2 ng/kg body weight/day with a 20,000-fold reduction, primarily due to concerns about immune system effects (EFSA, 2023).

In response to these restrictions, industries have increasingly substituted BPA with analogs like Bisphenol S (BPS) and Bisphenol F (BPF), now common in “BPA-free” products (Akash et al., 2023; Catenza et al., 2021; Maniradhan & Calivarathan, 2023; Martínez-Guijarro et al., 2024). However, BPS and BPF are also endocrine disruptors, with studies showing they can induce oxidative stress, DNA damage, and hormonal disruptions, potentially affecting reproductive, developmental, and metabolic systems (Ozyurt et al., 2023; Rochester & Bolden, 2015; Zhou et al., 2019). Moreover, some studies suggest that BPS and BPF may exhibit endocrine-disrupting potency comparable to, or even exceeding, that of BPA in certain biological systems (Pötzl et al., 2024; Rochester & Bolden, 2015). Their rising prevalence necessitates further investigation, especially in vulnerable populations like children.

The European Union has implemented regulations to limit BPA exposure, including bans on its use in baby bottles and food contact materials for infants (Usman & Ahmad, 2016). BPS and BPF are also under scrutiny for their potential risks (Mandel et al., 2019). In parallel, the Human Biomonitoring for Europe (HBM4EU) initiative has established Human Biomonitoring Guidance Values (HBM-GVs) for bisphenols to standardize risk assessment using urinary biomonitoring data (Correia-Sá et al., 2017; Garí et al., 2023; Ougier et al., 2021). Here physiologically based pharmacokinetic (PBPK) modeling has become an essential chemical risk assessment tool, allowing for the estimation of internal exposure from measured biomarker levels. For BPA, which is rapidly metabolized and excreted in urine, reverse dosimetry using PBPK principles offers a scientifically robust method to translate spot urine concentrations into estimates of daily intake (EFSA Panel on Food Contact Materials, Enzymes and Processing Aids (CEP) et al., 2023; Karrer et al., 2018; Ougier et al., 2021). The HBM-GV for BPA in children (135 ng/mL) was based on the 2015 EFSA TDI and PBPK modeling (Ougier et al., 2021). However, this value does not yet reflect the 2023 TDI reduction, highlighting the need for updated guidance values.

This study presents the first human biomonitoring assessment of BPA, BPS, and BPF in urine samples from Finnish children aged 3–6 years. To do this, we collected urine samples from children at daycare centers, assessed health risks by comparing urine bisphenol levels to HBM-GVs, estimated daily BPA intake via PBPK-based reverse dosimetry, and interpreted findings considering the revised 2023 EFSA TDI.

## Materials and methods

### Ethical permissions

Ethical approval was granted by the Tampere University Ethical Board (Statement 132/2022, Request 52/2022: "Evaluation and Reduction of New Persistent Organic Pollutants in Preschools of Finland"). All procedures were carried out in accordance with the ethical standards of the 1964 Declaration of Helsinki and its later amendments. Research permission was obtained from the City of Tampere to approach daycare centers. Informed consent was provided by parents of participating children.

### Selection of daycare centers and recruitment of children

Eighteen daycare centers in Tampere, Finland, were selected which represent diverse socioeconomic areas. Children aged 3–6 years attending daycare for more than 20 h per week. Parents received written invitations, and those who volunteered provided informed consent. They also completed a questionnaire on socioeconomic status, home characteristics, and children’s dietary habits and material use (e.g., plastics, Teflon, microwave use).

### Urine sample collection

Urine samples were collected from 40 children across 18 daycare centers in Spring 2023. Two to three children per daycare center participated. Parents collected their child’s first morning urine in a provided polypropylene tube and delivered it to the daycare center on a specified day. Samples were placed in insulated boxes with dry ice and transferred to a − 20 °C freezer within a few hours, where they were stored until analysis. For chemical analysis, frozen samples were shipped from Tampere University to the THL laboratory on dry ice, following standard procedures for transporting biological specimens.

### Chemical analysis

BPA, BPS, and BPF were analyzed at the Finnish Institute for Health and Welfare (THL) using a validated, accredited (evaluated and accepted by FINAS Finnish Accreditation Service in years 2013 for BPA and 2020 for BPS and BPF) procedure. Isotope-labeled internal standards (BPA-d6-glucuronide 8 ng, BPS-d8 8 ng, and BPF-d10 80 ng) were added to 0.40 mL urine samples. To measure total bisphenols (conjugated and non-conjugated), samples underwent enzymatic hydrolysis with β-glucuronidase/sulfatase (Helix pomatia H2; glucuronidase activity 304199 units/mL, sulfatase activity 2976 units/mL, Sigma-Aldrich) at 37 °C for 20 h. No buffer solutions were added into the samples. After hydrolysis, samples were diluted with 3 mL water, extracted with 3 mL ethyl acetate, evaporated to dryness under nitrogen gas, and reconstituted in 100 µL of 50% methanol. Calibration samples (*n* = 7) were prepared similarly to the real urine samples. Different calibration levels of all the analytes were prepared by spiking the respective native analyte concentration in the ultrapure water sample to achieve the final concentration range of 0.50–25 ng/mL for each bisphenol. Instrumental analysis was performed using a Thermo Scientific UltiMate 3000 Rapid Separation LC system coupled to a Thermo Finnigan TSQ Quantum Discovery MAX triple quadrupole mass spectrometer. LC–MS/MS parameters are detailed elsewhere (Rajasärkkä et al., 2014). The limit of quantification (LOQ) for all bisphenols, based on the lowest calibration point with acceptable signal/noise ratio (> 8) and precision, was 0.50 ng/mL and the calculated theoretical limit of detection (LOD, signal/noise ratio > 3) was 0.19 ng/mL.

For quality control, a procedural blank (ultrapure water matrix) and a G-EQUAS (the German External Quality Assessment Scheme) interlaboratory study urine sample were analyzed alongside the real urine samples using the same extraction and measurement procedure. The G-EQUAS control sample used was from Round 56/2015, with a target BPA concentration of 1.86 ng/mL and an acceptable range from 1.41 to 2.31 ng/mL as specified by the G-EQUAS organizer. Creatinine was not measured in this study; therefore, urinary concentrations are reported as non-creatinine-adjusted values.

All the samples were analyzed in a single sample batch. The levels of BPA, BPS and BPF in the blank sample were below the LOQ (as well as LOD), therefore a blank subtraction from the sample concentration had no effect on the results. In the G-EQUAS control sample, the analyzed concentration of 2.2 ng/mL was within the acceptable range given by the G-EQUAS organizer.

The analytical method used in this study has been accredited by FINAS (Finnish Accreditation Service) for BPA in 2013 and for BPS and BPF in 2020. The accreditation process included a full method validation; however, validation data are not reported here as methodological development was not an objective of the present study.

### Risk assessment

Health risks associated with bisphenol exposure were evaluated using human biomonitoring guidance values (HBM-GVs) established under the human biomonitoring for Europe (HBM4EU) initiative (Ougier et al., 2021). These values were derived from the 2015 European food safety authority (EFSA) temporary tolerable daily intake (t-TDI) for BPA (4 µg/kg body weight/day) using physiologically based pharmacokinetic (PBPK) modeling to estimate steady-state urinary total BPA concentrations consistent with chronic oral exposure at the t-TDI level (Karrer et al., 2018; Ougier et al., 2021). The HBM-GV for BPA in children is 135 ng/mL (total BPA in urine), while for BPS it is 1 ng/mL (Meslin et al., 2022); no HBM-GV is currently available for BPF.

Risk Characterization Ratios (RCRs) were calculated as the ratio of measured urinary bisphenol concentrations to the corresponding HBM-GV (Meslin et al., 2022):

$${\mathrm{RCR}}_{\mathrm{HBM}}=\frac{\mathrm{urinary}\;\mathrm{concentration}\;\left(\mathrm{ng}/\mathrm{mL}\right)}{\mathrm{HBM}-\mathrm{GV}\left(\mathrm{ng}/\mathrm{mL}\right)}$$

Using HBM-GV_BPA_ for children ​ = 135 ng/mL and HBM-GV_BPS_ = 1 ng/mL. No HBM-GV exists for BPF. An RCR > 1 indicates potential health concern. It should be noted that the HBM-GV of 135 ng/mL for BPA was derived from the 2015 EFSA t-TDI (4 µg/kg bw/day) using PBPK modeling (Ougier et al., 2021), as no formally adopted updated HBM-GV based on the 2023 EFSA TDI currently exists. A theoretical recalculation suggests an equivalent urinary threshold of approximately 1.4 ng/L for children under the 2023 TDI (Meslin et al., 2022), which is discussed further in Sect. "PBPK-based intake estimation and health risk assessment".

In addition, estimated daily intakes (EDIs) for BPA were derived from spot urine concentrations using a population-level conversion factor derived from PBPK modeling, as recommended by HBM4EU and described by Ougier et al. (Ougier et al., 2021). In their approach, PBPK simulations were used to model the steady-state relationship between oral BPA intake (corresponding to the t-TDI) and total BPA concentration in urine for a reference child (19 kg, aged ~ 5 years). This approach is designed for population biomonitoring studies using spot urine samples, where individual daily urine output is not measured. Specifically, they estimated that a BPA t-TDI of 4 µg/kg bw/day for a child would correspond to a total urinary BPA concentration of 135 ng/mL, using PBPK modeling. Thus, the EDI was estimated by scaling the observed spot urine concentration relative to this PBPK-derived benchmark:

$$\mathrm{E}\mathrm{D}\mathrm{I}\left(\mu \mathrm{g}/\mathrm{k}\mathrm{g}\;\mathrm{b}\mathrm{w}/\mathrm{d}\mathrm{a}\mathrm{y}\right)=\mathrm{U}\mathrm{r}\mathrm{i}\mathrm{n}\mathrm{a}\mathrm{r}\mathrm{y}\;\mathrm{B}\mathrm{P}\mathrm{A} \left(\mathrm{n}\mathrm{g}/\mathrm{m}\mathrm{L}\right)\times \left(4\;\mu \mathrm{g}/\mathrm{k}\mathrm{g}\;\mathrm{b}\mathrm{w}/\mathrm{d}\mathrm{a}\mathrm{y}135\;\mathrm{n}\mathrm{g}/\mathrm{m}\mathrm{L}\right)$$

EDIs were compared to both the 2015 EFSA t-TDI (4 µg/kg body weight/day) and the revised 2023 EFSA TDI (0.2 ng/kg body weight/day) to contextualize exposure levels. This integrated approach aligns with HBM4EU guidelines for translating biomonitoring data into intake estimates and risk assessments, providing a framework for interpreting urinary bisphenol levels in young children.

### Statistical analysis

Descriptive statistics (detection frequency, median, 95th percentile, range) were calculated for each bisphenol. Values below the LOQ (0.5 ng/mL) were reported as " < LOQ" and treated as left-censored data in statistical analyses. Due to the high proportion of values below the LOQ, medians and percentiles are reported in accordance with guidelines for censored data (Helsel, 2012). Associations between age and bisphenol detection were examined using the Mann–Whitney U test, comparing age distributions between children with detected (> LOQ) versus non-detected (< LOQ) concentrations. The non-parametric Mann–Whitney U test was chosen due to low detection frequencies and small sample sizes in the detected groups. Statistical analyses were conducted using RStudio (version 4.2.2).

## Results

### Urinary concentrations of BPA, BPS, and BPF

**Fig. 1:**
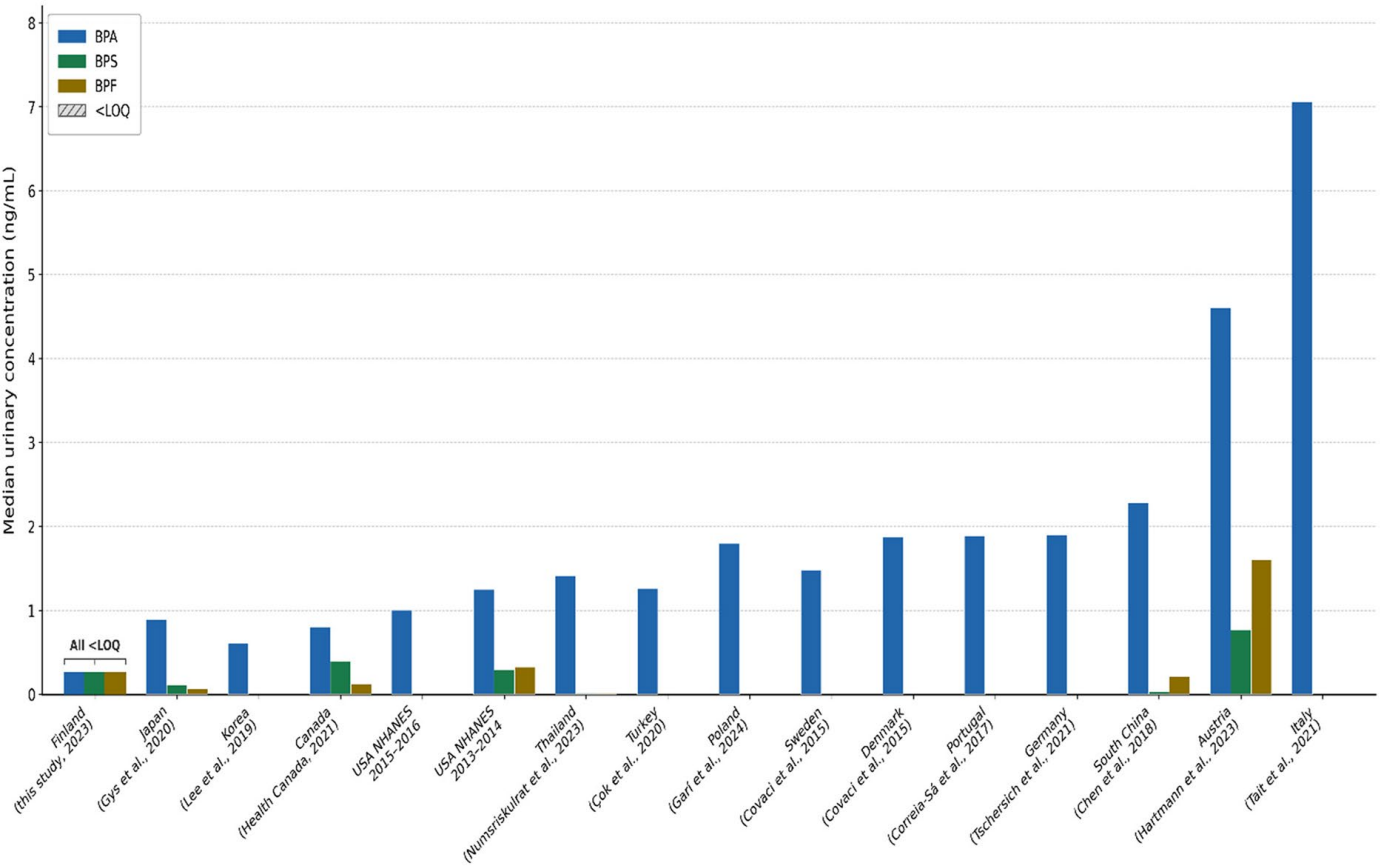
Global comparison of median urinary bisphenol concentrations (ng/mL) in children. BPA = bisphenol A; BPS = bisphenol S; BPF = bisphenol F. Studies are ordered by ascending BPA concentration. Hatched bars indicate values below the limit of quantification (<LOQ = 0.50 ng/mL), shown at LOQ/2 for visibility; all three bisphenols were <LOQ in Finland (this study). Empty bars indicate that the analyte was not reported in the respective study. Concentrations for Turkey (Çok et al., 2020) represent the mean of boys; Canada (Health Canada, 2021) and Thailand (Numsriskulrat et al., 2023) report geometric means

Urinary concentrations of bisphenol A (BPA), bisphenol S (BPS), and bisphenol F (BPF) were measured in 40 children aged 3–6 years. A visual comparison of these levels with global data is presented in Fig. 1.

Table 1 presents descriptive statistics for each compound, including detection frequency, median, 95th percentile, and range. No inferential statistics are reported for concentration distributions due to the high proportion of values below the LOQ**.**

**Table 1:** Urinary Concentrations of BPA, BPS, and BPF in Children Aged 3–6 Years (ng/mL)

Compound	Detection frequency (%)	Median (ng/mL)	95th percentile (ng/mL)	Range (ng/mL)
BPA	32.5	< 0.5	8.5	< 0.5–16
BPS	15	< 0.5	2.1	< 0.5–2.3
BPF	0	< 0.5	< 0.5	< 0.5

The 25th percentile is < LOQ for all analytes and is therefore not reported separately. LOD = 0.19 ng/mL; LOQ = 0.50 ng/mL for all compounds

### PBPK-based EDI for BPA and comparison to TDIs

Using PBPK reverse dosimetry, urinary BPA concentrations were translated into estimated daily intakes (EDIs) for a 5-year-old child, based on the reference model established by Ougier et al. (2021), in which the 2015 EFSA t-TDI of 4 µg/kg bw/day corresponds to a steady-state total urinary BPA concentration of 135 ng/mL. This PBPK-derived conversion factor is applied here as the scaling benchmark, since no formally adopted HBM-GV based on the 2023 EFSA TDI has yet been established by HBM4EU. Notably, a theoretical recalculation by Meslin et al., (2022) suggests that applying the 2023 TDI would yield an equivalent urinary threshold of approximately 1.4 ng/L for children — a value exceeded by all quantifiable BPA concentrations in our study. Table 2 shows EDIs calculated at the LOQ, P95, and maximum observed concentrations, compared against both the 2015 EFSA t-TDI (4 µg/kg bw/day) and the 2023 EFSA TDI (0.2 ng/kg bw/day), providing a full picture of risk under both regulatory frameworks. The implications of these comparisons are discussed further in Sect. "PBPK-based intake estimation and health risk assessment".

**Table 2:** Estimated daily intake (EDI) of BPA from urinary concentrations and comparison with EFSA guidance values

Urinary BPA (ng/mL)	EDI(µg/kg bw/day)*	EDI expressed as % of EFSA 2015 TDI	Exceedance relative to EFSA, 2023 TDI
LOQ (0.50)	0.0148	0.37%	74 x
P95(8.5)	0.252	6.3%	1,260 x
maximum (16)	0.474	11.9%	2,370x

Relative to the 2015 EFSA t-TDI, EDIs represented 0.37%–11.9% of the threshold, meaning all observed intakes were well below the earlier safety limit. In contrast, when compared to the much stricter 2023 EFSA TDI of 0.0002 µg/kg bw/day, all EDIs exceeded the threshold by factors ranging from 74 at the LOQ to over 2,300 at the maximum observed concentration.

### HBM-GV RCRs

Risk Characterization Ratios (RCRs) were derived by dividing measured urinary concentrations by the HBM-GVs (135 ng/mL for BPA; 1 ng/mL for BPS). No HBM-GV is available for BPF. Results are shown in Table 3**.**

**Table 3:** Risk Characterization Ratios (RCRs) for BPA and BPS relative to HBM-GVs

Analyte	HBM-GV (ng/mL)	P95 concentration (ng/mL)	RCR at P95	Maximum concentration (ng/mL)	RCR at maximum	% samples exceeding RCR > 1
BPA	135	8.5	0.063	16.0	0.118	0%
BPS	1	2.1	2.1	2.3	2.3	7.5%
BPF	-	< LOQ	-	< LOQ	-	-

Under current HBM-GVs (based on 2015 TDI) no exceedances were seen, but recalculation with the 2023 TDI would indicate exceedances at all levels. BPS showed exceedances in 3 out of 40 children (7.5%), with the maximum concentration of 2.3 ng/mL corresponding to an RCR of 2.3 considering the existing HBM-GV. BPF could not be assessed since all samples were < LOQ and no HBM-GV exists.

### Urinary bisphenol levels by sex, age, and exposure factors

The study included 18 boys and 22 girls. By age at time of sampling, 13 were aged 3 years, 14 aged 4 years, 8 aged 5 years, and 1 aged 6 years. BPA was detected in 35.3% of boys and 23.8% of girls; BPS in 17.6% of boys and 14.3% of girls; BPF was not detected in either sex. The Mann–Whitney U test was selected due to the small sample size and non-normal distribution of detected values; however, the low number of detections limits the strength of any conclusions drawn from these comparisons. No significant age differences were found between detected and non-detected groups for BPA (U = 126.0, p = 0.66) or BPS (U = 77.5, p = 0.27). For BPF, all concentrations were below the LOQ, precluding any age analysis.

Also, Material use (e.g., plastic container or microwave use) and dietary variables showed no associations with urinary bisphenol levels, likely due to the high proportion of non-detect values.

## Discussion

### Urinary concentrations of BPA, BPS, and BPF

This study presents the first human biomonitoring data on urinary concentrations of bisphenol A (BPA), bisphenol S (BPS), and bisphenol F (BPF) in Finnish children, aged 3–6 years. Given the endocrine-disrupting properties of bisphenols and children's susceptibility to such exposures (Lucarini et al., 2020), these data contribute valuable insight into environmental health risks in early life. In our study, BPA and BPS were detected in a subset of urine samples, while BPF was not detected in any participant. These findings are consistent with prior European studies reporting higher detection frequencies for BPA and BPS than for BPF (Gys et al., 2021; Vaccher et al., 2022).

### Comparison with global studies

We compared these findings with global studies in Table 4 and Fig. 1, which shows median urinary bisphenol concentrations across countries. Urinary BPA and BPS concentrations in our sample appear lower than those reported in several earlier studies conducted in other countries, including Italy, Germany, and Poland (Garí et al., 2024; Tait et al., 2021; Tschersich et al., 2021). Similarly, our levels are lower than those reported in more recent studies from Austria (Hartmann et al., 2023) and the US NHANES studies from 2013–2016 (EPA ACE Indicator B11, n.d.); Lehmler et al., 2018), which showed median BPA concentrations of 4.6 ng/mL and 1.0–1.25 ng/mL, respectively. Our findings are more comparable to recent Canadian data (Health Canada, 2021, 2024), which reported median BPA concentrations of 0.80 ng/mL in 3–5 year olds. However, such comparisons should consider contextual differences. Notably, the LOQ for BPF in the present study (0.50 ng/mL) is below all median BPF concentrations reported in the comparison studies included in Table 4, with the exception of Hartmann et al. (2023), who reported exceptionally high median BPF levels (1.6 ng/mL). This observation places the 0% detection frequency for BPF in context: it cannot be attributed to an insufficiently sensitive method, but rather reflects genuinely low or absent BPF exposure in this Finnish child population.

**Table 4:** Global comparison of urinary bisphenols in children

Country	year	n	age group	Urinary Bisphenols level
Finland (This study)	2023	40	3–6 years	BPA: < 0.50–16 ng/mL, median < 0.50 ng/mL BPS: < 0.50–2.3 ng/mL, median < 0.50 ng/mL BPF: < 0.50 ng/mL, median < 0.50 ng/mL
Austria (Hartmann et al., 2023)	2020	85	6–10 years	BPA: median 4.6 ng/mL BPS: median 0.77 ng/mL BPF: median 1.6 ng/mL
USA (NHANES) (Lehmler et al., 2018)	2013–2014	868	6–17 years	BPA: median 1.25 ng/mL BPS: median 0.29 ng/mL BPF: median 0.32 ng/mL
USA (NHANES) (EPA ACE Indicator B11, n.d.)	2015–2016	-	6–17 years	BPA: median 1.0 ng/mL
Canada (Health Canada, 2021, 2024)	2018–2019	-	3–5 years	BPA: Geometric mean 0.80 ng/mL BPS: Geometric mean 0.39 ng/mL BPF: Geometric mean 0.12 ng/mL
Italy (Tait et al., 2021)	2015–2017	900	4–14 years	BPA: median 7.06 ng/mL
Thailand (Numsriskulrat et al., 2023)		358	6–13 years	BPA: Geometric mean 1.41 ng/mL BPS: Geometric mean 0.014 ng/mL BPF: Geometric mean 0.013 ng/mL
Poland (Garí et al., 2024)		250	7 years	BPA: median 1.8 ng/mL
Turkey (Çok et al., 2020)	2015–2016	125	3–6 years	BPA: mean boys (1.26 μg/g creatinine) and mean girls (2.24 μg/g creatinine)
Germany (Tschersich et al., 2021)	2014–2017	515	3–17 years	BPA: Geometric mean 1.90 ng/mL
Japan(Gys et al., 2020)	2012_2017	396	7 years	BPA: median 0.89 ng/mL; BPS: median 0.11 ng/mL; BPF: median 0.07 ng/mL |
Portugal (Correia-Sá et al., 2017)	2014–2015	110	4–18 years	BPA: median 1.89 ng/mL
Denmark (Covaci et al., 2015)	2011–2012	142	6–11 years	BPA: Geometric mean 1.87 ng/mL
Sweden (Covaci et al., 2015)	2011–2012	97	6–11 years	BPA: Geometric mean 1.48 ng/mL
South China (Y. Chen et al., 2018)	2015	283	3–11 years	Mean BPA 2.28, 0.37 ng/mL Mean BPS 0.03, 0.07 ng/mL Mean BPF 0.21, 0.19 ng/mL
Korea (Lee et al., 2018)	2001–2006	164	3–5 years 7–9 years	BPA: median 0.76 μg/g creatinine BPA: median 0.61 μg/g creatinine overall: 0.61(ng/mL)
Guangzhou, China (Li et al., 2013)	2013	56	3–6 years	BPA: adjusted least square geometric mean (LSGM) 1.80 (ng/mL)

Many of the referenced studies were conducted a decade earlier, between 2011 and 2017, whereas our samples were collected in 2023. Since then, BPA use has been increasingly restricted in the EU, including limitations in food contact materials (Usman & Ahmad, 2016), which may explain the relatively lower concentrations observed here.

Additionally, our study focused exclusively on 3–6 years old children, while many earlier studies included a broader age range extending into adolescence (Correia-Sá et al., 2017; Covaci et al., 2015). Older children have different behavior patterns, including more independent dietary choices and use of personal care or electronic products, which could influence bisphenol exposure. Thus, both regulatory timing and differences in age structure are important factors when interpreting cross-country comparisons.

### PBPK-based intake estimation and health risk assessment

To estimate external BPA exposure from urinary concentrations, we applied a reverse dosimetry approach grounded in physiologically based pharmacokinetic (PBPK) principles, consistent with EFSA and HBM4EU recommendations (Garí et al., 2023). Interestingly, in 4 out of 6 cases where BPS was measurable, BPA concentrations remained below the LOQ, suggesting that exposure sources for these compounds may not fully overlap. As shown in Table 2, all EDIs exceeded the revised 2023 EFSA TDI of 0.2 ng/kg bw/day, even at concentrations near the analytical LOQ. It should be noted that the LOQ for BPA in the present study (0.50 ng/mL) substantially exceeds the theoretical urinary threshold of approximately 1.4 ng/L corresponding to the 2023 EFSA TDI, making direct compliance assessment through routine biomonitoring practically impossible. The elevated LOQ for BPA is largely a consequence of background BPA contamination from labware during the dedicated matrix clean-up procedure, a well-recognised challenge in BPA biomonitoring. Importantly, while BPA LOQs are typically higher than those for alternative bisphenols in most studies, the LOQs for BPA, BPS, and BPF are identical in the present method (0.50 ng/mL). It can be expected that as general BPA exposure and environmental background contamination decrease, achievable LOQs will also decline.

Risk characterization using HBM-GVs provided a somewhat different perspective. None of the measured BPA concentrations exceeded the currently established HBM-GV of 135 ng/mL, which is derived from the 2015 EFSA TDI (Ougier et al., 2021). However, as theoretically recalculated by Meslin et al., (2022), applying the 2023 TDI would yield an equivalent urinary HBM-GV of approximately 1.4 ng/L for children — a threshold exceeded by all quantifiable BPA concentrations in our study, even those near the LOQ. This discrepancy emphasizes the urgent need to update HBM-GVs in accordance with the most recent EFSA risk assessment to ensure consistency between toxicological benchmarks and biomonitoring practice. For BPS, the maximum urinary concentration (2.3 ng/mL) exceeded its HBM-GV of 1 ng/mL, resulting in an RCR > 1 in 7.5% of children. Although detections were infrequent, these exceedances suggest that BPS exposure is relevant for risk assessment in early childhood. By contrast, BPF was not detected in any participant, and in the absence of an HBM-GV, no risk characterization could be performed.

More refined approaches such as quality assurance procedures, repeated sampling to reduce day-to-day variability, and advanced PBPK models that incorporate age-specific metabolism and excretion may be needed to place such low-level findings into context.

### Limitations, and future directions

This study integrates PBPK-informed reverse dosimetry to interpret biomonitoring data in Finnish children (3–6 years), leveraging current toxicological benchmarks (e.g., EFSA's, 2023 BPA TDI) and aligning with European practices. The use of first-morning urine samples is preferred for enhanced practicality, while the simplified PBPK approach provided a transparent framework for estimating internal exposure despite lacking route-specific or individual metabolic data.

Key limitations include the small sample size (n = 40) and single urine collection, which may underestimate daily exposure variability. The absence of an HBM-GV for BPF precluded its risk assessment, and low detection frequencies limited subgroup analyses. The PBPK model assumes steady-state conditions and uniform excretion and does not account for non-urinary excretion or metabolic variability, necessitating caution in generalizability.

Urinary concentrations were not corrected for dilution (e.g., by creatinine or specific gravity). This limits direct comparability with studies reporting creatinine-adjusted concentrations. A refractometer for specific gravity measurement could address this limitation in future studies and would greatly improve comparability of results.

A potential analytical limitation is the LOQ of 0.50 ng/mL applied to all three bisphenols. For BPS, this exceeds median concentrations reported in some studies (e.g., 0.11 ng/mL in Gys et al., 2020; 0.014 ng/mL in Numsriskulrat et al., 2023), which may have resulted in some BPS-exposed children being classified as non-exposed. The absence of BPF detections also limits conclusions regarding its exposure or health relevance in this population. Future studies using methods with lower LOQs would help better characterise exposure to these BPA alternatives in young children. Despite these limitations, the use of accredited laboratory methods and PBPK-based scaling enhances the robustness of exposure interpretation.

Future work should prioritize larger, diverse cohorts with longitudinal sampling to capture exposure dynamics. Establishing pediatric HBM-GVs for BPS and BPF and integrating multi-pathway exposure data would strengthen risk assessments. Repeated sampling and more detailed exposure data could further support the application of full PBPK models to improve intake estimation.

## Conclusion

This study presents the first biomonitoring data on BPA, BPS, and BPF in urine samples from Finnish children. BPA and BPS were detected in a subset of participants, while BPF was not quantifiable. PBPK-based intake estimates for BPA exceeded the revised 2023 EFSA TDI, even at concentrations near the analytical LOQ. At the same time, none of the concentrations exceeded the currently established HBM-GV for BPA, which is derived from the earlier 2015 EFSA TDI. This highlights the importance of updating HBM-GVs to reflect the most recent EFSA evaluation.

For BPS, the maximum concentration exceeded its HBM-GV, while no updated EFSA benchmark is yet available for this compound. These results indicate that both BPA and its analogues remain relevant for exposure assessment in early childhood and highlight the value of continued biomonitoring in this age group.

## Data Availability

The datasets analyzed during the current study are available from the corresponding author on reasonable request.
